# Combining pharmacophore- and MD-based modelling for phase II metabolism prediction

**DOI:** 10.1186/1758-2946-6-S1-O15

**Published:** 2014-03-11

**Authors:** Christin Rakers, Gerhard Wolber

**Affiliations:** 1 Computer-Aided Drug Design, Pharmaceutical and Medicinal Chemistry, Institute of Pharmacy, Freie Universität Berlin, 14195 Berlin, Germany

## 

As metabolism is considered a main cause for adverse drug reactions and failures of new drug candidates, our goal is to establish an *in silico* method to efficiently predict phase II metabolism – in particular sulfotransferase (SULT) activity. Since sulfotransferases exhibit low substrate specificities caused by their high degree of conformational freedom [1], activity prediction is a challenging task.

We therefore established a workflow based on molecular dynamics (MD) simulations to cover the whole spectrum of structural flexibility and incorporated it into multiple pharmacophores that represent specific modes of action. Using an ensemble of pharmacophores for virtual screening ensures accurate categorization of potential SULT ligands (e.g. substrates, inhibitors). Recent advances in MD technology [2] allowed for refinement of these pharmacophores by high-throughput MD simulations of ligand-target complexes. In addition, the initial binding process of a soluble ligand to the substrate-binding site of SULT was captured in unbiased 100 ns simulations using the software Desmond [3].
